# Cocaine- and amphetamine-regulated transcript promotes the differentiation of mouse bone marrow-derived mesenchymal stem cells into neural cells

**DOI:** 10.1186/1471-2202-12-67

**Published:** 2011-07-14

**Authors:** Zhuo Liu, Danqing Huang, Meijuan Zhang, Zhibin Chen, Jiali Jin, Siyuan Huang, Zhuo Zhang, Zhongyuan Wang, Lei Chen, Ling Chen, Yun Xu

**Affiliations:** 1 Department of Neurology, Drum Tower Hospital of Nanjing Medical University, 321 Zhongshan Road, Nanjing, Jiangsu, 210008, PR. China; 2 Department of Neurology, Affiliated Drum Tower Hospital of Nanjing University Medical School, 321 Zhongshan Road, Nanjing, Jiangsu, 21008, PR. China; 3 Department of Neurology, Jiangsu Province Geriatric Hospital, 65 Jiangsu Road, Nanjing, Jiangsu, 210024, PR. China; 4 The State Key Laboratory of Pharmaceutical Biotechnology, Nanjing University, 22 Hankou Road, Nanjing, Jiangsu, 210093, PR. China; 5 Jiangsu Key Laboratory for Molecular Medicine, Nanjing University, 22 Hankou Road, Nanjing, Jiangsu, 210093, PR China; 6 Department of Physiology, Nanjing Medical University, 140 Hanzhong Road, Nanjing, Jiangsu, 210029, PR China

## Abstract

**Background:**

Neural tissue has limited potential to self-renew after neurological damage. Cell therapy using BM-MSCs (bone marrow mesenchymal stromal cells) seems like a promising approach for the treatment of neurological diseases. However, the neural differentiation of stem cells influenced by massive factors and interactions is not well studied at present.

**Results:**

In this work, we isolated and identified MSCs from mouse bone marrow. Co-cultured with CART (0.4 nM) for six days, BM-MSCs were differentiated into neuron-like cells by the observation of optical microscopy. Immunofluorescence demonstrated that the differentiated BM-MSCs expressed neural specific markers including MAP-2, Nestin, NeuN and GFAP. In addition, NeuN positive cells could co-localize with TH or ChAT by double-labled immunofluorescence and Nissl bodies were found in several differentiated cells by Nissl stain. Furthermore, BDNF and NGF were increased by CART using RT-PCR.

**Conclusion:**

This study demonstrated that CART could promote the differentiation of BM-MSCs into neural cells through increasing neurofactors, including BNDF and NGF. Combined application of CART and BM-MSCs may be a promising cell-based therapy for neurological diseases.

## Background

Mesenchymal stem cells (MSCs) are attractive for the regeneration of damaged tissues in clinical applications, since they are characterized as undifferentiated cells, able to self-renew with a high proliferative capacity and possess mesodermal differentiation potential [1]. Bone marrow-derived MSCs have great potential as therapeutic agents for neurological diseases, because they are easily obtained from bone marrow and expand rapidly *in vitro*. Moreover, there is a lower risk of rejection using MSCs compared to other sources of stem cells as they can be autogenic. It has been held that MSCs can give rise to osteocytes, chondrocytes, adipocytes, and neural cells [2,3]. However, current induction methods obtain a lower percentage of neural differentiation and consume long induction time.

Cocaine- and amphetamine-regulated transcript (CART) peptides have emerged as major neurotransmitters and hormones. They are widely distributed in the CNS and involved in regulating many processes, including food intake, the maintenance of body weight and endocrine functions. Moreover, previous study demonstrated that CART was up-regulated in the cerebral cortex following focal cerebral ischemia *in vivo *and in cultured cortical neurons subjected to oxygen-glucose deprivation (OGD) in vitro. This regulation led to the reduction of infarct size and OGD-induced cell death [4]. Also, CART promoted the survival and differentiation of primary hippocampal neurons by up-regulating BDNF mRNA expression and protein synthesis [2].

NGF is critical for the survival and maintenance of sympathetic and sensory neurons. Without it, these neurons undergo apoptosis [3]. Nerve growth factor induces axonal growth including axonal branching and a bit of elongation [5]. BDNF is the second neurotrophic factor to be characterized after NGF. They help support the survival of existing neurons and encourage the growth and differentiation of new neurons and synapses [6,7]. Emerging evidence established that growth factors such as BDNF and NGF are physiological inductors for neural differentiation of MSCs not only in the MSCs, recipients but also in the MSCs, co-cultured medium [8,9].

This study tested the hypothesis that CART could promote the differentiation of MSCs into neural cells through increasing neurofactors such as BNDF and NGF.

## Results

### 1. Identification of mouse MSCs

**Figure 1:**
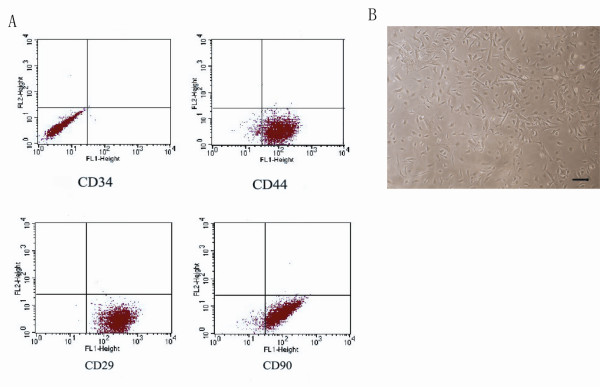
**Identification of MSCs *in vitro***. Identification of BM-MSC *in vitro*. (A) The fluorescent activated cell sorting analysis showed that the adherent cells were positive for the well-defined MSC surface markers including CD29, CD44, and CD99, while negative for CD34. CD34 precluded the origination of hematopoietic progenitors. (B) MSCs extracted from the mouse bone marrow presented the fibroblastic shape with dark centers as well as transparent peripheries by the observation of phase contract invert microscopy (scale bar = 50 uM).

Firstly, cell morphology was observed daily by phase contrast invert microscopy. In the early days, individual adherent cells appeared. Among the adherent cells, some were fibroblastic in shape and the others were round with dark centers and transparent peripheries. In the subsequent days, some fibroblastic cells proliferated. They gave rise to clones of pure fibroblastic cells, each of which was composed of several cells. Finally, these cells were almost completely obscured by the fibroblastic cells (Figure 1B). Fluorescent activated cell sorting (FACS) analysis demonstrated that the expanded plastic adherent cells were positive for the mesenchymal stem cell-associated surface markers CD29, CD44 and CD99, but negative for the hematopoietic progenitor's specific surface maker CD34 (Figure 1A). Thus, the cells used in this study fulfilled all criterion to be defined as MSCs.

### 2. Neuronal induction of mouse MSCs treated by CART

#### 2.1 Cell Culture

**Figure 2:**
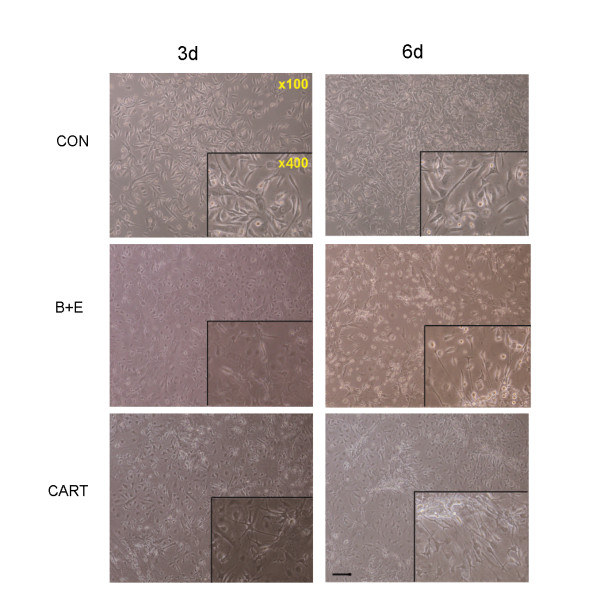
**Morphological change of MSCs with or without the exposure to CART**. Particular changes in morphology happened to MSCs with the exposure to CART. Similar to the situation of the bFGF/EGF-treated group, several MSCs incubated with CART for 3 days became shorter and nucleus-convergent. They subsequently evolved to display round cell bodies with long axons in 6 days. In the control group, mesenchmal stem cells nearly kept stable in the appearance within the 6 days of, observation (scale bar = 50 uM).

48-72 h after induction, some cells became shorter. In addition, the cytoplasm gathered towards the nucleus and formed the axons and dendrites (Figure 2). Another 3 days later, the majority of cells turned into neuron-like cells. They had large and round cell bodies with longer axons similar to the shape of cells induced by endothelial growth factor (EGF) and basic fibroblast grow factor (bFGF). The uninduced group remained in fiber-like cells (Figure 2).

#### 2.2 Immunofluorescence Assay of neural marker proteins

**Figure 3:**
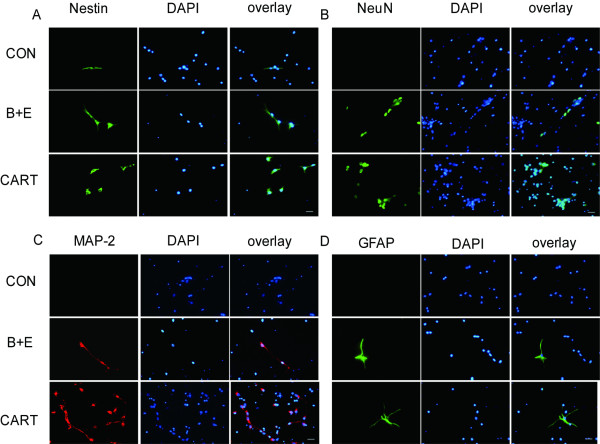
**Differential spectrum of MSCs determined by Immunofluorescence**. Nestin, GFAP, MAP-2 and NeuN were involved to detect neural progenitors, glial cells, and mature neurons. In the CART treated group (A), the percentage of Nestin (green) positive cells occupied 25.4 ± 2.1% in Day 3 and 47.1 ± 1.9% in Day 6. (B) The NeuN (green) positive rate was 32.1 ± 2.3% in 3 days and 40.3 ± 2.7% in 6 days. The differentiation ratio resembled that of bFGF/EGF. (C) MAP-2 (red) was detected in 30.8 ± 4.7% and 41.2 ± 3.1% of all cells in Day 3 and Day 6. (D) GFAP (green) ranks 20.5 ± 2.5% in 3 days and 21.3 ± 2.2% in 6 days. The converted rate resembled that of bFGF/EGF. The untreated group was used as a control..

MSCs treated or not treated by CART were prepared for immunofluorescence analysis by antibodies against the neural precursor Nestin (green, Figure 3A), the markers of neurons MAP-2 (red, Figure 3C) and NeuN (green, Figure 3B), and the marker of astrocytes GFAP (green, Figure 3D). Quantification of immunofluorescence staining from three independent experiments revealed that Nestin, MAP-2, GFAP and NeuN were 25.4 ± 2.1%, 30.8 ± 4.7%, 20.5 ± 2.5%, 32.1 ± 2.3% of all cells in 3 days and 47.1 ± 1.9%, 41.2 ± 3.1%, 21.3 ± 2.2%, 40.3 ± 2.7% in 6 days (Table 1), respectively. A similar rate of conversion was induced by EGF/bFGF, with 27.1 ± 2.0%, 29.3 ± 3.7%, 11.4 ± 2.6%, 31.1 ± 2.9% in 3 days and 37.1 ± 2.9%, 33.2 ± 3.0%, 14.8 ± 4.2%, 39.3 ± 3.8% in 6 days. Only a trace amount of MSCs in the untreated group expressed low levels of Nestin (8.2 ± 2.2% in 3 days, 13.1 ± 3.0% in 6 days) but no MAP-2, GFAP or NeuN.

**Table 1:** Differential spectrum of MSCs determined by Immunofluorescence

		Nestin	MAP-2	GFAP	NeuN
3d	CON	8.2 ± 2.2%	2.7 ± 0.8%	1.4 ± 0.3%	3.1 ± 1.2%
	CART	25.4 ± 2.1%*	30.8 ± 4.7%*	20.5 ± 2.5%*	32.1 ± 2.3%*
	B+E	27.1 ± 2.0%	29.3 ± 3.7%	11.4 ± 2.6%	31.1 ± 2.9%

6d	CON	13.0 ± 3.0%	2.9 ± 1.0%	1.8 ± 0.5%	3.6 ± 1.1%
	CART	47.1 ± 1.9%*	41.2 ± 3.1%*	21.3 ± 2.2%*	40.3 ± 2.7%*
	B+E	38.3 ± 2.9%	33.2 ± 2.8%	14.8 ± 4.2%	39.3 ± 3.8%

* p < 0.05 versus control group

#### 2.3 Function of these differentiated cells

**Figure 4:**
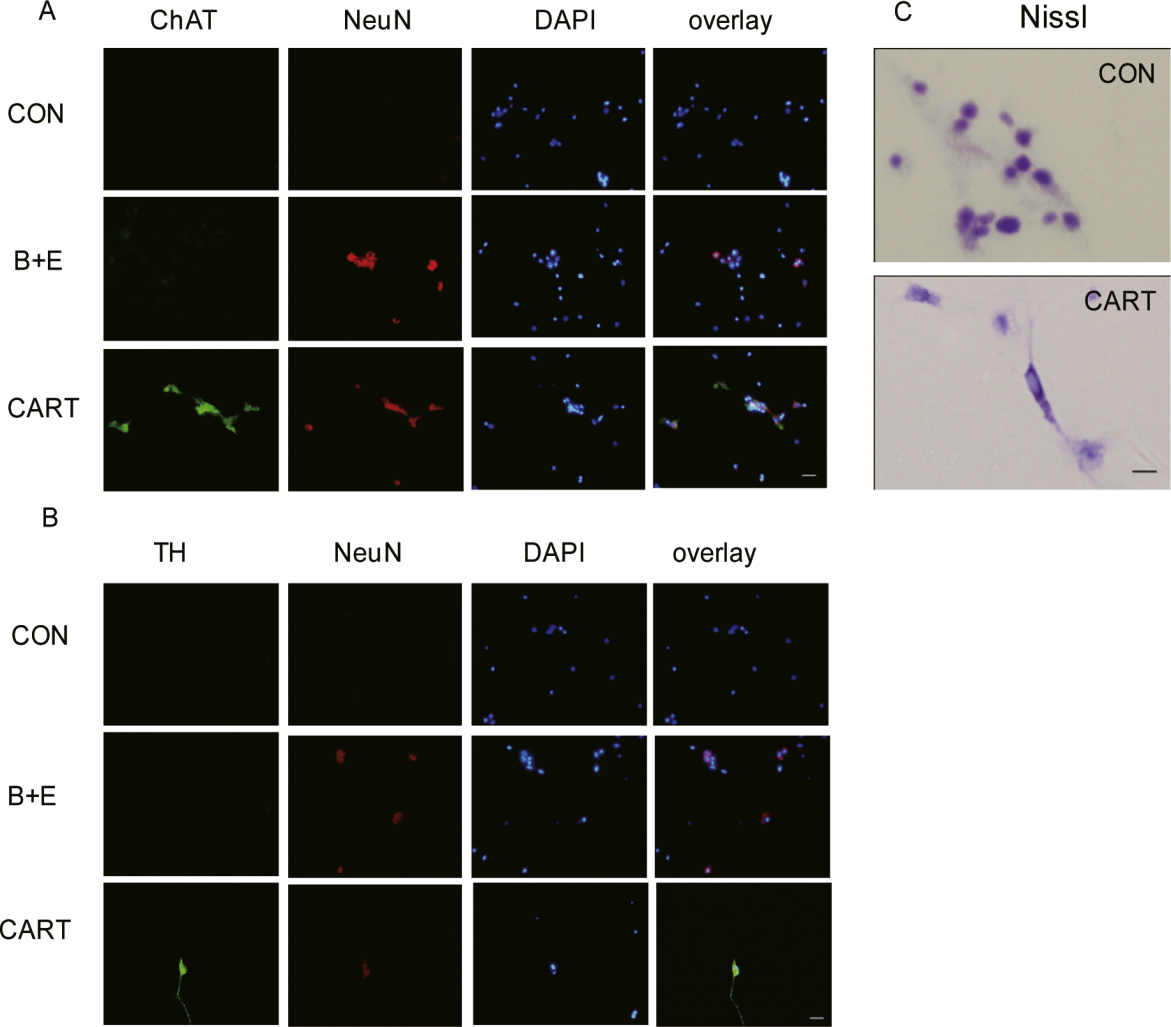
**Founctional test of the differentiated MSCs**. Cholinergic neurons and dopaminergic neurons were investigated by double labeled immunofluorescence with the antibodies against ChAT (green) and NeuN as well as TH (green) and NeuN (red) with the purpose of finding founctional neurons. NeuN positive cells co-expressed with ChAT (A) or TH (B) in the CART-treated group and the bFGF/EGF-incubated group. (C) The transferred neurons exhibited cytoplasmic dark blue praticles and light blue nucleus by Nissl stain. MSCs devoid of CART displayed dark blue nucleus and light blue cytoplasm (scale bar = 50 uM).

To investigate the function of differentiated neurons, ChAT (specific surface expression in cholinergic neurons) and TH (marker of dopaminergic neurons) were detected by immunofluorescence. Results showed that a few NeuN positive cells co-expressed ChAT (Figure 4A) or TH (Figure 4B). The MSCs in the control and the growth factor-treated groups hardly expressed either of them. Also, we carried out the Nissl stain to look for Nissl bodies among the MSCs with CART for 6 days. We found that Nissl bodies existed as dark blue particles in the cytoplasm of several differentiated MSCs (Figure 4C). No similar structure was detected in control group. What is more, excitable properties of MSC-derived neural like cells were detected by whole cell recording. Voltage-gated potassium current and voltage-gated calcium current were elicited in CART treated MSCs (Additional file 1).

### 3. CART up-regulated neurofactors BDNF and NGF

**Figure 5:**
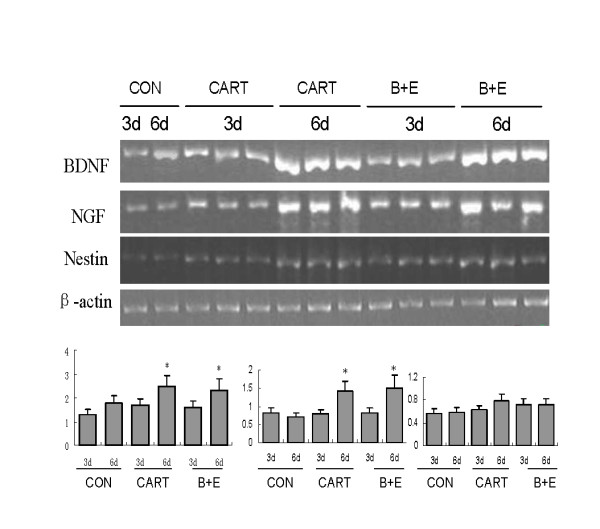
**Neurotrophic factors effected by CART**. RT-PCR was performed to calculate the endogenous expression of BDNF and NGF in Day 3 and Day 6. In contrast with the control group, the amount of BDNF was 1.68-fold and 2.45-fold higher with the incubation of CART at 3 days and 6 days. NGF was also higher with incubation of cart, with 1.05-fold of the control group in Day 3 and 2.01-fold of the control group in Day 6. There were no significant differences between CART treated-group and growth factor treated-group. (* p < 0.05 versus control group).

In addition to immunofluorescence assay, the neural specific RNA transcripts BDNF and NGF were amplified by RT-PCR. For each marker, the RNA extracts of MSCs were used as controls. This study showed that BDNF and NGF were highly induced on 6 days after CART treatment. Compared to the control group, the amount of BDNF was 1.68 folds (p > 0.05) and 2.45 folds (p < 0.05) higher with the incubation of CART 3 days and 6 days. NGF was also higher with the incubation of CART, with 1.05 fold of control group in Day 3 (p > 0.05) and 2.01 folds of control group in Day 6 (p < 0.05). There were no significant differences between CART treated-group and growth factor treated-group (Figure 5).

## Discussion

In the present study, we thoroughly assessed for the first time the capacity of CART to enhance the neural differentiation of MSCs. We demonstrated that, when exposed to CART, MAP-2, GFAP or NeuN positive cells were traced in the cultured MSCs. Furthermore, the proposed differentiated neurons not only have the potency to express ChAT and TH, but also display the Nissl bodies in the cytoplasm. In addition, neurotrophic factors including NGF and BDNF were up-regulated in CART-treated group. Our results suggest that CART may promote the differentiation of MSCs *in vitro *by enhancing the endogenous expression of NGF and BDNF.

MSCs extracted from mouse bone marrow have great multiplication potency. Cell-doubling time is 48-72 h and cells can be expanded in culture for more than 60 doublings [10]. Herein, we isolated BM-MSCs with the characteristics involving adherence to cell culture plastic, specific surface antigen expression, and multipotent differentiation which met the criterion defined by the Mesenchymal and Tissue Stem Cell Committee of the International Society for Cellular Therapy [11]. Also, BM-MSCs possess pluripotency defined by their ability to differentiate into bone, fat, cartilage and muscle [1,12-14]. Compelling evidences unveiled that MSCs are competent to break germ layer commitment and differentiate into cells expressing neuroectodermal properties [15]. Such a capacity opens extensive possibilities for autologous therapeutic treatments in a variety of neurological disorders. Also, as there are ethical issues related to isolating stem cells from fetal tissue and from adults, alternative sources of neuron-like cells for cell therapy are necessary. Indeed, a portion of studies indicate that, in contrast to the primary stem cells, the transplantation of pre-differentiated stem cells can diminish the risk of tumogenesis in the host patients [16]. However, current inducing methods obtain a lower percentage of neuronal differentiation by a long inducing time [17]. In 2000, Sanchez-Ramos *et al *used RA in combination with BDNF to introduce differentiation of BMSC to neural phenotypes. NeuN and GFAP positive cells only rank 0.5 and 1% respectively [18]. Likewise, Woodbury *et al *found β-mercaptoethanol (BME) can rapidly induce the transition from MSCs into neurons but not glial cells [19]. There is also a debate about whether or not the converted neural phenotyped cells are able to work [20]. This premise provided the impetus to investigate better and new reagent which can yield functional neuronal cells at a high rate.

Here we extended our study on the neuroectodermal conversion of BM-MSCs *in vitro *by exposure to the prospective peptide CART. After treatment with CART, we found that CART could successfully differentiate MSCs into neural cells *in vitro*. These cells were morphologically similar to neural stem cells. More importantly, they could express neural progenitor stem cell marker Nestin and mature glial marker GFAP as well as mature neuron markers like MAP-2 and NeuN. It indicated that the protocol of using CART for three or six days induced significant changes in morphology and expression of markers of early and mature neural cell types. Similar with induction rate of bFGF plus EGF, CART exposure obtained that MAP-2, GFAP and NeuN expressions were found in 30.8 ± 4.7%, 20.5 ± 2.5%, 32.1 ± 2.3% of all cells in 3 days, and 41.2 ± 3.1%, 21.3 ± 2.2%, 40.3 ± 2.7% in 6 days (Figure 4). However, the expression of one or even two neuronal proteins does not prove that the cell bearing these "neuronal markers" is capable of all the complex functions of a neuron. It will be important to determine whether these cells possess functional characteristics of neurons. Subsequently, double-labeled immunofluorescence was performed to calculate the function of the converted cells. It was intriguing that the differentiated NeuN positive cells co-expressed ChAT or TH. Our study on excitable property of MSC-derived neural like cells demonstrated that the differentiated cells pocess founctional patassium and cacium channels (Additional file 1). Recent research discovered that the neuron-like cells differentiated from BM-MSCs resemble the endogenous neural progenitors in morphological, immunocytochemical, and functional characteristics [21]. Another independent investigator also reported that the transformed BM-MSCs exhibit both neuron-like biochemical function and some corresponding electrophysiological properties [22].

CART is an endogenous neuropeptide which is widely distributed in the brain and peripheral nervous system. It has been well-established that CART can promote the survival and differentiation of neurons. Some investigators disclosed that CART has the capability to modulate the expression of growth factors and neurotrophic factors, which probably participated in the underlying effect of CART [23]. Based on this theory, we made an assumption that neurotrophic factors could be the bridge between CART and stem cell differentiation. In order to verify this hypothesis, the amount of NGF and BDNF was determined by RT-PCR. The result that neurotrophic factors are significant higher in CART treated group implied CART could promote the endogenous production of neurotrophic factors. Neurotrophic factors in cell and tissue culture have been shown to promote neuronal survival and differentiation and also regulate the growth of axons and dendrites [24,25]. The presence of growth factors such as BDNF or NGF in the transplantation or the co-culture experiment are considered as physiological inductors for neural differentiation of MSCs [8,9]. MSCs can induce a variety of neuro-regulatory proteins in addition to BDNF and β-NGF [26]. Further study demonstrated that the NGF and BDNF receptor TrkA and TrkB do exist on the surface of MSCs [27]. Moreover, the TrkB receptor modified by BDNF may trigger the MAPK signal pathway. This process contributes to the maturation and neural differentiation of MSCs [20]. Early studies confirmed that BDNF+RA (retinoic acid) adult bone marrow stromal cells can be induced to differentiate into neural cells *in vitro *[28]. However, CART stimulated the endogenous BDNF and NGF expression of MSCs. This stimulation may imitate the natural physiological pattern. As a result, we obtained a higher yield rate than in previous studies. In the hippocampus, BDNF and other neurotrophins are expressed from early stage of neuron development [29,30]. This process is a prerequisite for the differentiation of neurons derived from hippocampal precursor cells *in vitro *[31-33]. Overall, findings suggest that the BDNF and NGF enhanced by CART may be the foundation of stem cell differentiation and maturation.

## Conclusion

In summary, we identified CART as a potential neurotrophic factor. CART can promote the neural differentiation of BM-MSCs, probably through enhancing the endogeneous expression of neurotrophic factors. Combined our previous studies [4,34], this study further confirmed the benefitial role of CART in the central nervous system. Besides the protective effect on neurons through complicated interaction with immunology and inflammation, CART also contributes to neuronal cells differentiation and generation. In the future, CART pre-treatment may become an attractive and implemental method in stem cell therapy for neurological disease.

## Methods

### Experimental animals and CART

Kunming mice (three-week old; (20 ± 2) g) were from the animal center of the Nanjing University of China. All mice were housed under a 12-hour light/dark cycle with food and water provided. The Animal Care and Use Committee of the Nanjing University approved all mouse protocols. CART was from Phoenix Pharmaceuticals, Inc. (USA).

### MSCs isolation, purification and culture

Mice were killed by cervical dislocation, and bone marrow samples were collected from tibias and femurs for MSCs. MSCs were isolated according to the method described by Pittenger *et al *[1]. Mononuclear cells were resuspended in Dulbecco's Modified Eagle's Medium (DMEM, Gibco, USA), 20% fetal bovine serum (FBS, Sigma, USA), 100 U/ml penicillin, and 100 μg/ml streptomycin. About 1 × 10^6 ^cells were plated into 25 cm^2 ^flasks (Corning, USA) and incubated at 37°C with 5% CO_2 _and 95% relative humidity. The medium was first changed after 72 hours and then every 3 to 4 days thereafter. When the cultures reached approximately 90% of confluence, MSCs were recovered by the addition of 0.25% trypsin-ethylenediamine tetraacetic acid (EDTA) solution (Invitrogen, USA) and replated into passage culture at 1:3. The purity of MSCs was over 95% after 20 days from initial seeding.

### Characterization of isolated MSCs

MSCs have the following characteristics: adherence to tissue culture plastic and several relatively specific cell surface markers [11]. The morphology of plastic adherent cells were monitored by an inverse microscope (TS100, Nikon, Japan). Cell surface molecules was analyzed on passage 3 cultures of MSCs using flowcytometry and the following procedures. Cells were detached from the flask by incubation with a solution of 0.25% trypsin-EDTA for 5 min at room temperature. They were then recovered by centrifugation and washed in flowcytometry buffer consisting of 2% BSA and 0.1% sodium azide (Sigma) in PBS. They were separated into aliquots of 1 × 10^6 ^cells. Next, they were incubated with conjugated monoclonal antibodies, either CD34 and CD44 or CD29 and CD90 (Becton Dickinson, USA), for 20 min. Washed cells were then incubated with secondary antibodies (Alexa-594 conjugated donkey anti-rabbit, 1:50 Jackson, USA) for 20 min in the dark. After washing, the cells were resuspended in 500 μl PBS and analyzed using a FACSCalibur software (Becton Dickinson, USA).

### Neuronal Induction

Subconfluent passage 3 cultures of MSCs were maintained in DMEM/20% FBS.

To initiate neuronal differentiation, the cells were washed with PBS and transferred to neuronal induction media composed of DMEM/**0.4n**M CART (Phoenix Pharmaceuticals, Inc.) at time ranging from 3 days to 6 days. As a positive control, MSCs were differentiated by DMEM/10 ug.ml^-1^EGF/10 ug.ml^-1^bFGF(Sigma, USA). At the end of the cultivation period, the cells were photographed and then fixed with frozen pyruvate for 10 min, preparing for the immunofluorescence assay. The cells were also used for RNA extraction and reverse transcription-polymerase chain reaction (RT-PCR) analysis of neurogenic gene expression.

### Immunofluorescence Assay and Nissl stain

MSCs were plated on the matrigel-coated dishes (FISHER, USA). The MSCs fixed by frozen pyruvate were permeabilited for 30 min in PBS containing 0.1% Triton X-100. The samples were incubated with following primary antibodies at 4°C overnight: antibodies for Nestin (1: 50, Sigma, USA), MAP-2 (1:100, Sigma, USA), GFAP (1:100, Sigma, USA), NeuN (1:100, Sigma, USA), ChAT (1:50, Sigma, USA), and TH(1: 100, Singma, USA). Primary antibodies were detected by using Cy2- or Cy3- conjugated goat anti-rabbit or anti-mouse secondary antibodies (1: 200, Sigma, USA) for 1 hour at room temperature. After reaction with secondary antibodies, the cells were stained with 100 nM DAPI (Sigma, USA) for 15 seconds, then mounted. Fluorescence-labeled MSCs were viewed and photographed (AX10, ZEISS, German).

To detect Nissl bodies, differentiated cells were harvested and fixed in 4% neutral buffered formalin for 1 h, and then stained for 30 min with 0.1% Cresyl violet in room tempreture.

### Real-time quantitative PCR

At 3 or 6 days, post-induction, the MSCs reached approximately 80% of confluence, and total RNA was extracted with Trizol (Invitrogen, USA) according to the manufacturer's instructions. cDNA was synthesized from RNA using the AMV reverse transcriptase (Gibco, USA). Real-time quantitative PCR was performed with a 7500 real-time PCR System (ABI 7500, USA). After initial denaturation, PCR was carried out for 30 cycles at 94°C for 45 seconds and 56°C for 45 seconds. Relative abundance of mRNA was calculated after normalization to GAPDH ribosomal RNA. The following primers were designed and used for real-time PCR:

BDNF: forward 5' ATG CTC AGC AGT CAA GTG CC 3', reverse 5' AGT AAG GGC CCG AAC ATA CG 3'

NGF: forward 5' AGC CCA CTG GAC TAA ACT TCA G 3', reverse 5' CAA AGG TGT GAG TCG TGG TGC A 3'

### Statistical Analysis

Differences among treatment groups were analyzed with a t-test for two groups (SPSS 13.0, USA). The criterion for statistical significance was set at *P *< 0.05. All values are reported as the mean ± SEM.

## List of abbreviations

BM-MSC: bone marrow mesenchymal stromal cell; CART: cocaine- and amphetamine-regulated transcript; OGD: oxygen-glucose deprivation; BDNF: brain derived neurotrophic factor; NGF: nerve growth factor; bFGF: basic fibroblast grow factor; EGF: endothelial growth factor; ChAT: cholineacetyltransferase; TH: tyrosine hydroxylase

## Authors' contributions

YX designed the experiments and edited this paper. ZL, DQH, LC carried out the experiments. ZBC did data analysis and made figures. MJZ and SYH helped to write the paper. ZZ and ZYW participated in part of the experiments. All authors read and approved the final manuscript.

## Supplementary Material

Additonal File 1 **Excitable properties of MSC-derived neural like cells**. (A) Voltage-gated potassium current was evoked by a series of depolarizing pulses from -100 mV to +50 mV stepping by 10 mV with interval time of 5 sec. (B) voltage-gated calcium current was elicited by depolarizing to -40 mV (200 ms) from a holding potential of -80 mV and then further depolarized to 0 mV (200 ms) with interval time of 5 sec. Click here for file
