# Supplementary figures and images for: Cocaine- and amphetamine-regulated transcript promotes the differentiation of mouse bone marrow-derived mesenchymal stem cells into neural cells
Source: BMC Neurosci. 2011 Jul 14;12:67. doi: 10.1186/1471-2202-12-67 (PMC3199873; doi:10.1186/1471-2202-12-67)

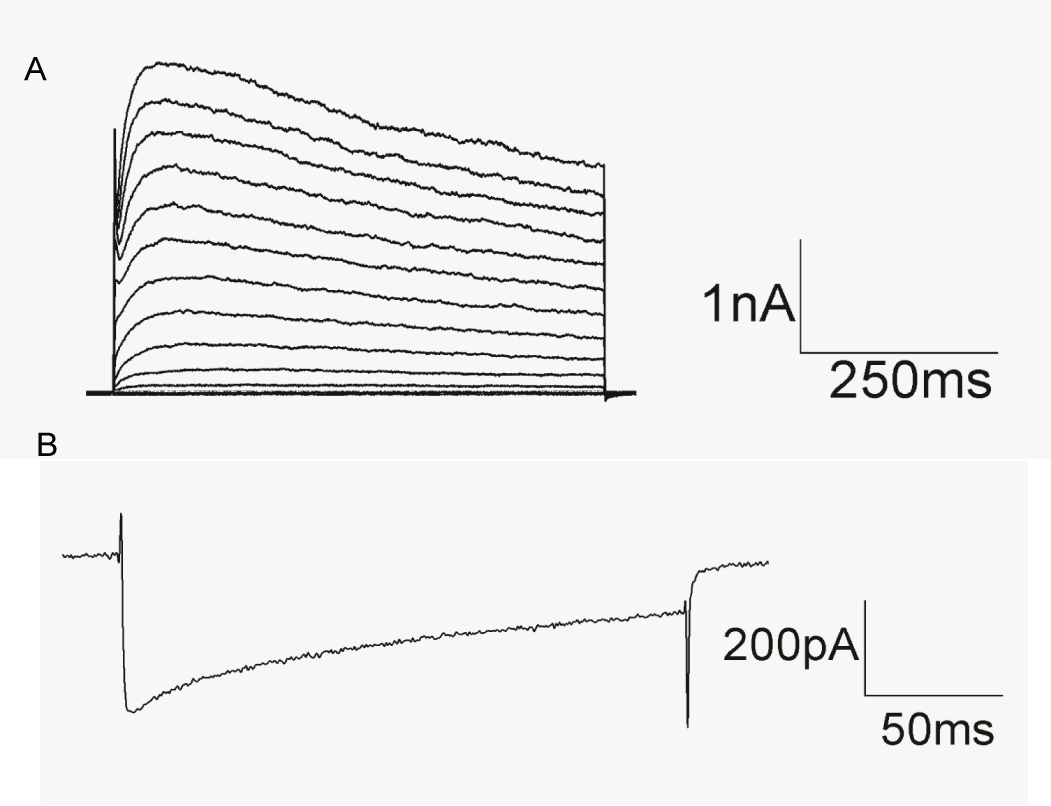

Supplement: Additonal File 1 — Excitable properties of MSC-derived neural like cells. (A) Voltage-gated potassium current was evoked by a series of depolarizing pulses from -100 mV to +50 mV stepping by 10 mV with interval time of 5 sec. (B) voltage-gated calcium current was elicited by depolarizing to -40 mV (200 ms) from a holding potential of -80 mV and then further depolarized to 0 mV (200 ms) with interval time of 5 sec. [file 1471-2202-12-67-S1.TIFF]
